# Construction of Lunar Soil Simulants-Based Aluminum-Ion Battery Systems

**DOI:** 10.3390/ma18030471

**Published:** 2025-01-21

**Authors:** Shaokang Su, Jingzhen Li, Chunhao Sun, Kai Du, Chengjie Wang, Mingshan Han, Jing Geng, Yongde Long, Yuxiang Hu

**Affiliations:** Key Laboratory of Advanced Functional Materials of Education Ministry of China, College of Materials Science and Engineering, Beijing University of Technology, Beijing 100124, China; ssk610050375@163.com (S.S.); jzli11@bjut.edu.cn (J.L.); chunhaosun9808@163.com (C.S.); dukai@emails.bjut.edu.cn (K.D.); wangchengjie@emails.bjut.edu.cn (C.W.); mshan@emails.bjut.edu.cn (M.H.); b202377028@emails.bjut.edu.cn (J.G.); yongde_long@bjut.edu.cn (Y.L.)

**Keywords:** lunar soil simulants, in situ resource utilization, ilmenite, aluminum-ion batteries

## Abstract

With the development of space technology, in situ resource utilization (ISRU) of lunar resources holds great potential for constructing lunar bases. This study, for the first time, proposes the in situ construction of lunar soil simulants-based battery systems. When novel ilmenite cathode materials are applied in aqueous aluminum-ion batteries (AAIBs), a facile ball milling treatment is used to simulate the natural characteristics of lunar-based ilmenite with proper electrochemical performance. The in situ constructed lunar soil-based batteries demonstrated a practical capacity of 68.1 mAh g^−1^ at 1.0 A g^−1^ with a capacity retention rate of 89.6% after 100 cycles. Even at a high current density of 5.0 A g^−1^, the as-prepared batteries still maintained a capacity of 41.7 mAh g^−1^. This study provides a promising energy storage solution for lunar bases and promotes sustainable energy technologies through in situ utilization of lunar resources.

## 1. Introduction

As Earth’s resources become increasingly depleted and environmental issues increase, the scientific community is actively exploring outer space and resource utilization to ensure the long-term sustainability of humanity. In this context, significant progress has been made in manned space exploration, particularly in lunar exploration, which has garnered widespread attention [1,2,3,4]. The development of long-term life support systems on the Moon not only holds significant technological importance but also carries profound scientific and economic value. The effective utilization of lunar resources offers a potential solution to Earth’s resource shortages, while also driving the extension of human civilization into outer space.

Lunar soil, as one of the most abundant resources on the Moon, has attracted increasing scientific attention in recent years. Current research primarily focuses on the development of lunar soil as functional materials, such as building materials. For example, scientists have successfully extracted aluminum, fibers, and iron from lunar soil, and have used high-temperature vacuum sintering techniques to directly produce lunar soil bricks [5,6,7,8]. Additionally, the recent discovery of crystal water resources in the lunar regolith provides new possibilities for the further utilization of lunar resources [9,10]. While initial studies on energy conversion using lunar soil have made progress, as lunar exploration shifts toward long-term habitation, the development of efficient energy storage systems has become a critical issue [11]. Note that transporting existing battery systems from Earth to the Moon is extremely costly. For instance, the cost of transporting materials equivalent to the weight of a bottle of water to the Moon can reach approximately USD 200,000 [12]. Therefore, the ability of in situ resource utilization (ISRU) lunar materials to construct required equipment is of paramount importance.

In existing ISRU technologies, considerable research has proposed utilizing thermoelectric conversion devices or solar cell for power generation on the Moon [13,14]. Nonetheless, these solutions have evident limitations. The Moon’s day–night cycle is exceptionally long, with each phase lasting about 14 Earth days. Due to the prolonged lunar nights, such energy generation systems are unable to meet the energy demands during the extended dark periods. Additionally, the Moon’s extreme temperature variations between day and night mean that solar energy, while highly efficient during the lunar day, cannot provide stable power during the long lunar nights. As a result, existing energy storage systems fail to address the critical challenge of storing energy for the lunar nighttime. This highlights the need for the development of an energy storage device that can be widely applicable both during the day and at night. Inspired by previous in situ utilization technologies, we considered whether it would be feasible to directly use lunar soil to construct energy systems, such as batteries [15]. However, the elemental composition of lunar soil is significantly different from the materials commonly used in terrestrial batteries, making it necessary to develop specialized battery systems suited to the lunar environment [16,17]. Mature technologies such as lithium-ion and lead–acid batteries are not feasible for lunar applications, as lunar soil lacks critical elements such as lithium and lead [18,19,20,21]. Moreover, the conventional lithium-ion batteries could pose safety risks in the Moon’s extreme environment, where high radiation and temperature fluctuations could lead to thermal runaway, endangering both equipment and personnel issues [22,23]. Therefore, the in situ fabrication of high-safety battery systems using lunar resources has become a significant issue. Inspired by the previous exploration on extracted aluminum, high-safety aluminum-based batteries would be suitable candidates for lunar batteries [24,25,26,27,28]. Meanwhile, through in-depth analysis of lunar soil samples, we found that lunar soil is rich in ilmenite (FeTiO_3_), which has been extensively studied as an electrode material in various battery systems, showcasing its significant potential as a highly adaptable and efficient electrode material. This analysis makes ilmenite a particularly promising candidate for cathode material in lunar-based aluminum batteries [29,30,31,32].

Herein, we propose, for the first time, a high-safety lunar soil simulants-based battery system, consisting of an ilmenite cathode and aluminum anode. This approach not only significantly reduces spacecraft load and launch costs but also avoids excessive consumption of Earth’s resources. The newly developed ilmenite-based aluminum battery exhibits proper electrochemical performance, achieving a practical capacity of 68.1 mAh g^−1^ at a high current density of 1.0 A g^−1^ with a capacity retention rate of 89.6% after 100 cycles. This innovative design provides a reliable energy solution for long-term lunar habitation, while greatly advancing the development and utilization of lunar resources, holding significant scientific and economic value.

## 2. Experimental Section

### 2.1. Sample Preparation

Simulative ilmenite (FeTiO_3_) powder was purchased from Aladdin Industrial Corporation Co., Ltd. (Shanghai, China). and stored in Ar-filled glovebox ([H_2_O], [O_2_] < 0.01 ppm). To simulate the lunar soil simulants, the as-prepared sample was ball-milled in a planetary ball mill at room temperature under an air atmosphere for 36 h at a speed of 500 rpm. The weight ratio of the powder to the milling balls was set at 1:20. The FeTiO_3_ powder was placed in a milling jar and sealed with an O-ring. After milling, the sample was washed with ethanol, dried overnight under vacuum, and then ground to obtain the desired black FeTiO_3_ powder.

### 2.2. Materials Characterization

The crystal structures of FeTiO_3_ were analyzed using X-ray diffraction (XRD) within a range of 10° to 80°, utilizing a Bruker D8 instrument (Bruker, Bremen, Germany) with Cu Kα radiation at 40 kV and 40 A. The microstructure of the sample was observed through high-resolution transmission electron microscopy (HRTEM) using a Titan G300 (Thermo Fisher Scientific, Hillsboro, OR, USA, 300 eV), which was also equipped with an electron diffraction spectroscope (EDS) for element distribution mapping. Selected area electron diffraction (SAED) patterns were obtained using a Talox F200 at 200 kV (Thermo Fisher Scientific, Hillsboro, OR, USA). Morphological characterizations were conducted with a scanning electron microscope (SEM JEOL 7001 F, JEOL, Ltd., Tokyo, Japan). X-ray photoelectron spectroscopy (XPS) using an ESCALAB 250Xi (Thermo Fisher Scientific, Waltham, MA, USA) was employed to analyze the element valence states. Additionally, Raman spectra were collected with a Renishaw inVia spectrometer (Renishaw, Wotton-under-Edge, UK) using a 532 nm laser.

### 2.3. Electrochemical Measurements

The CR2032 coin cells were assembled at room temperature (25 °C) using FeTiO_3_ as the cathode, high-purity aluminum as the anode, and Whatman glass fiber as the separator to evaluate their electrochemical performance. Initially, FeTiO_3_, Ketjenblack (KB), and polyvinylidene fluoride (PVDF) were combined in a weight ratio of 6:3:1 in N-methyl-2-pyrrolidone (NMP) to create a uniform slurry. This mixture was applied to a 10 mm diameter titanium foil with a loading mass of approximately 0.13 ± 0.03 mg cm^−2^, then dried in a vacuum oven at 90 °C for 10 h. The cells were then assembled in air using 1.0 M Al(OTF)_3_ electrolyte for further testing. Their constant current discharge/charge performance was studied at ambient temperature within a voltage range of 0.30–1.70 V using a NEWARE multi-channel battery testing system. Additionally, cyclic voltammetry (CV) curves were recorded on a CHI760E electrochemical workstation with a scan rate of 0.5 mV s^−1^ across a voltage range of 0.01–1.70 V.

## 3. Results

**Scheme 1:**
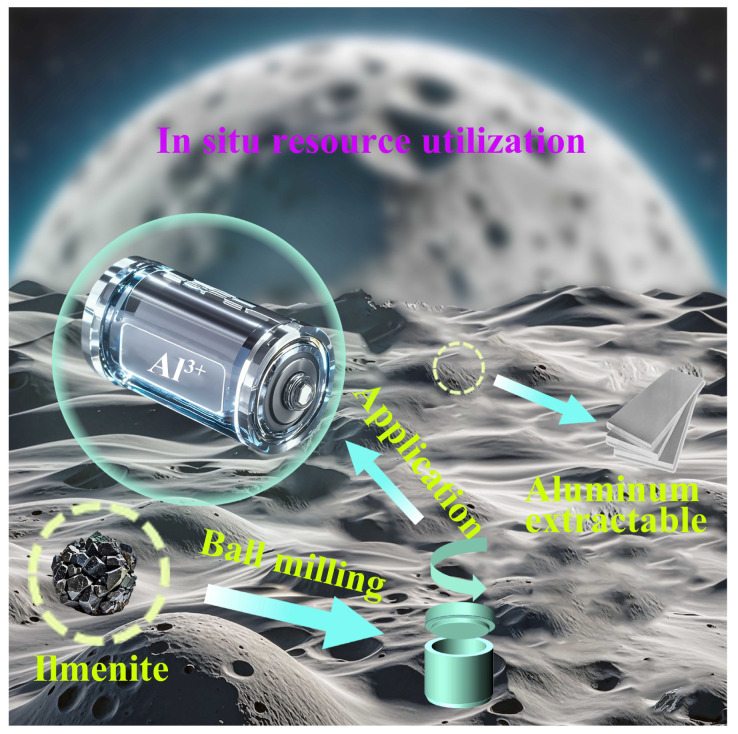
Schematic illustration of an in situ fabricated aluminum-ion battery based on lunar soil resources, such as ilmenite.

As shown in the Scheme 1, the design concept of in situ battery fabrication using lunar soil resources is presented. High-purity aluminum metal can be extracted as the anode material through high-temperature electrolysis of certain raw materials from the lunar soil [24]. Additionally, water/crystal water resources found in the lunar regolith can be extracted in situ and used for aqueous batteries [9,10]. Furthermore, lunar soil contains abundant ilmenite (FeTiO_3_, around 17.8%) [33,34], which would be promising cathode material in aqueous aluminum-ion battery systems.

**Figure 1:**
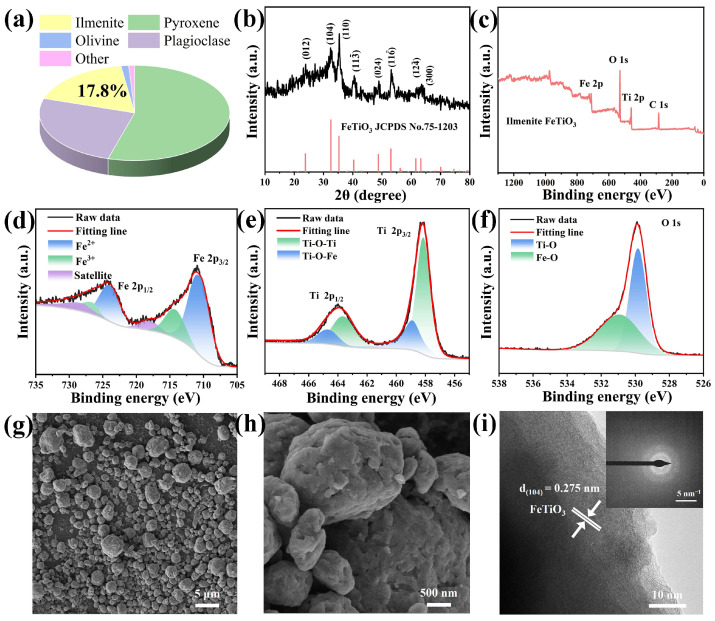
Physical characterizations of lunar soil simulants, such as FeTiO_3_. (**a**) Compositional content in Moon Sea basalt (vol %: Pyroxene: 54.5%, Plagioclase: 25.4%, Ilmenite: 17.8%, Olivine: 1.3%, Other: 1%). (**b**) Crystal structure of FeTiO_3_. (**c**) X-ray photoelectron spectroscopy (XPS) survey spectrum of FeTiO_3_. (**d**–**f**) High-resolution XPS spectra of Fe 2p, Ti 2p, and O 1s for FeTiO_3_. (**g**,**h**) Scanning electron microscopy (SEM) images of FeTiO_3_ at different magnifications. (**i**) Transmission electron microscopy (TEM) and selected area electron diffraction (SAED) images of FeTiO_3_.

Ilmenite is primarily derived from mare basalts, one of the most widely distributed rock types on the lunar surface, originating from large-scale volcanic activity during the Moon’s early history [35]. Remote sensing data and lunar sample analyses reveal that mare basalts cover approximately 17% of the lunar surface. In addition to ilmenite, these basalts are composed of pyroxene, olivine, and plagioclase, among other minerals (Figure 1a). The abundance of ilmenite makes it a critical target for lunar resource development. However, due to the lack of an atmosphere and global magnetic field on the Moon, lunar soil has been exposed to extreme conditions such as solar wind, cosmic rays, and micrometeorite impacts, leading to significant space weathering. The unique environment on the Moon causes the microstructure of lunar soil minerals to differ markedly from those on Earth. Prolonged space weathering results in the refinement and surface modification of lunar soil particles. Specifically, under the continuous bombardment of high-energy particles and micrometeorites, lunar soil particles undergo mechanical fragmentation and surface sputtering, gradually becoming more refined, usually reaching submicron or even nanoscale sizes [36,37]. To simulate the characteristics of ilmenite in lunar soil, the initial FeTiO_3_ samples were subjected to 36 h of ball milling. To verify the crystalline structure of the milled samples, X-ray diffraction (XRD) analysis was conducted on the as-prepared simulative sample. As shown in Figure 1b, clear diffraction peaks were observed in the 10° to 80° range, indicating that the FeTiO_3_ particles retained good crystallinity. The diffraction peaks at 23.8°, 32.5°, 35.2°, 40.3°, 48.7°, 53.0°, 61.5°, and 63.3° correspond to the (012), (104), (110), (11$\bar{3}$), (024), (11$\bar{6}$), (12$\bar{4}$) and (300) crystal planes of FeTiO_3_ (JCPDS No.75-1203), respectively. The average crystallite size, calculated from the XRD data using the Scherrer equation, is around 17.6 nm. This relatively small crystallite size suggests that the material possesses a fine microstructure, which would contribute to its enhanced electrochemical performance in aluminum-ion batteries.

To further determine the surface elemental composition and chemical environment of the FeTiO_3_ samples, X-ray photoelectron spectroscopy (XPS) was performed. The survey spectrum (Figure 1c) confirmed the presence of Ti, Fe, and O elements. The high-resolution XPS spectrum of Fe (Figure 1d) revealed three pairs of characteristic peaks corresponding to Fe^2+^ in Fe 2p_3/2_ and Fe 2p_1/2_ (711.0 eV and 724.2 eV), Fe^3+^ in Fe 2p_3/2_ and Fe 2p_1/2_ (714.5 eV and 726.9 eV), and satellite peaks (718.2 eV and 728.5 eV). The XPS spectrum of Ti (Figure 1e) indicated two distinct chemical environments: Ti-O-Fe with peaks at 458.9 eV and 464.8 eV, and Ti-O-Ti with peaks at 458.1 eV and 463.7 eV, corresponding to Ti 2p_3/2_ and Ti 2p_1/2_, respectively. Additionally, the O 1s spectrum of FeTiO_3_ (Figure 1f) displayed two fitted peaks at 530.9 eV and 529.8 eV, corresponding to Fe-O and Ti-O bonds [38,39,40].

Detailed morphological and structural characterization of FeTiO_3_ was conducted using scanning electron microscopy (SEM) at different magnifications. The SEM images of the pre-milled FeTiO_3_ (Figure S1) displayed a distinct blocky structure. Although the material exhibited good crystallinity (Figure S2), most particles were within the micron scale, with irregular sheet-like morphologies. The particle surfaces were relatively smooth, and there were significant size differences and apparent voids between particles. After ball milling, the microstructure of FeTiO_3_ underwent substantial changes. As shown in Figure 1g, the particle size significantly decreased, and the morphology became more uniform, exhibiting regular spherical or near-spherical shapes. The uniformity of the particles improved notably, with tighter contact between particles, forming a more compact microstructure. This structure contributes to the formation of an effective conductive network, thereby enhancing the overall conductivity of the electrode. Figure 1h further reveals the surface details of the milled particles. The surface roughness and micro-porous structure of the milled particles were evident, which helps increase the material’s specific surface area, providing more active sites for electrochemical reactions. Additionally, the micro-defects or roughness on the material’s surface may alleviate the stress caused by volume expansion, thereby extending the battery’s cycle life. The smaller particle size and enriched surface microstructure can significantly shorten the ion diffusion path, facilitating the rapid intercalation and deintercalation of aluminum ions during charge–discharge cycles, thus improving the battery’s rate performance. Energy Dispersive X-ray Spectroscopy (EDS) analysis of the SEM images (Figure S3) further confirmed the elemental composition of FeTiO_3_, showing the presence of Fe, Ti, and O, which were consistent with the expected composition, supporting the structural integrity for energy storage applications.

In the transmission electron microscopy (TEM) images (Figure S4), the overall morphology of the ball-milled FeTiO_3_ particles can be clearly observed. The particle edges were sharp, and the shapes tended to be more regular, which is consistent with the SEM results. This result indicates that ball milling effectively reduced and homogenized the larger blocky particles, not only successfully simulating the structural state of FeTiO_3_ particles on the lunar surface but also favorably enhancing the material’s electrochemical performance. The high-resolution TEM images (Figure 1i) further demonstrated the crystalline structure of the FeTiO_3_ material. Clear lattice fringes were observed, with a lattice spacing of 0.275 nm, corresponding to the (104) crystal plane of FeTiO_3_. The selected area electron diffraction (SAED) pattern in the inset displayed bright diffraction rings, further confirming the high crystallinity of the FeTiO_3_ material.

**Figure 2:**
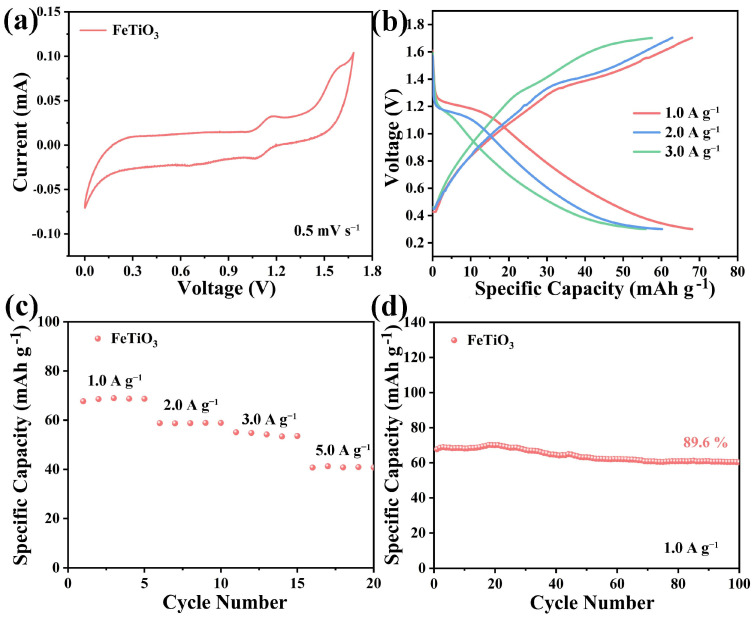
Electrochemical properties of lunar soil simulate-based batteries performance. (**a**) Cyclic voltammetry (CV) curves of FeTiO_3_ at 0.5 mV s^−1^. (**b**) Galvanostatic charge and discharge (GCD) curves at 1.0, 2.0, and 3.0 A g^−1^. (**c**) Rate capability of FeTiO_3_ at various current densities ranging from 1.0 to 5.0 A g^−1^. (**d**) Long-term cycling performance of FeTiO_3_ at a current density of 1.0 A g^−1^.

To further explore the potential of simulative FeTiO_3_ material in aluminum-ion storage, we have pioneeringly applied FeTiO_3_ as a cathode material in aqueous aluminum-ion batteries (AAIBs), and conducted a series of electrochemical tests. Figure 2a shows the cyclic voltammetry (CV) curves of FeTiO_3_ in AAIBs, with a testing voltage range of 0.01 to 1.70 V. The curves reveal prominent redox peaks at approximately 1.1 V and 1.2 V, which are directly related to the intercalation and deintercalation processes of aluminum ions in FeTiO_3_. The small voltage difference between the redox peaks indicates that this electrode material has low polarization and good electrochemical activity. The galvanostatic charge–discharge (GCD) curves demonstrate that FeTiO_3_ maintains a high discharge plateau (approximately 1.2 V) and a specific capacity of 68.1 mAh g^−1^ at a current density of 1.0 A g^−1^ (Figure 2b). Figure 2c and Figure S5 illustrate the rate performance of the FeTiO_3_ electrode at different current densities. As the current density increases from 1.0 A g^−1^ to 5.0 A g^−1^, the specific capacity of the electrode slightly decreases but remains at approximately 41.7 mAh g^−1^, indicating that the material has good capacity retention at high rates. This enhanced rate performance reflects the rapid diffusion of aluminum ions within the electrode material and the effective (de)intercalation ability, further demonstrating the potential of this material in AAIBs. After 100 cycles, the battery’s capacity retention rate reached 89.6% (Figure 2d), indicating that FeTiO_3_ maintains good structural stability and electrochemical performance over multiple cycles. Particularly in the early stages of cycling, the electrode material exhibited minimal capacity decay, demonstrating its superior ability to maintain structural integrity during repeated aluminum-ion (de)intercalation processes. SEM image of the FeTiO_3_ electrode after cycling was added to evaluate its structural stability (Figure S6). This long cycle life is crucial for practical applications, ensuring the stability and reliability of the battery during prolonged use. Even at a high current density of 5.0 A g^−1^, FeTiO_3_ still retained 79.4% of its capacity (Figure S7). To investigate the changes in the electrochemical performance of the battery during cycling, electrochemical impedance spectroscopy (EIS) measurements were performed before and after cycling. Figure S8 shows the Nyquist plots of the battery before and after cycling, where the semicircle in the high-frequency region corresponds to the charge transfer resistance (R_ct_), and the inclined line in the low-frequency region reflects the ion diffusion impedance. After cycling, R_ct_ decreased significantly, indicating that the electrode interface underwent optimization during the cycling process, resulting in an improved charge transfer process and enhanced electrochemical activity.

To further understand the electrochemical performance of the proposed lunar soil simulants-based aluminum-ion battery system, the charge and discharge mechanisms were analyzed through the corresponding reaction equations at the cathode and anode. During the discharge process, Al^3+^ intercalate into the FeTiO_3_ cathode. This intercalation leads to the reduction of Fe^3+^ to Fe^2+^, as described by the following reaction:

FeTiO_3_ + xAl^3+^ + 3xe^−^ → Al_x_FeTiO_3_

At the aluminum anode, oxidation occurs, releasing aluminum ions into the electrolyte:

Al → Al^3+^ + 3e^−^

During the charging process, these reactions are reversed, with aluminum ions deintercalating from the cathode and aluminum metal being deposited at the anode. The overall reaction mechanism ensures the reversibility and stability of the system. It is important to note that the high charge density and large ionic radius of trivalent aluminum ions can limit the performance of conventional cathode materials in AAIBs, leading to lower specific capacity and rate performance. In this context, the FeTiO_3_ cathode material in AAIBs not only ensures high safety but also exhibits favorable electrochemical performance. Additionally, to investigate the electrochemical kinetics of the FeTiO_3_ electrode, CV tests were performed at scan rates ranging from 0.2 to 1.0 mV s^−1^ (Figure S9). The peak current (i) and scan rate (*v*) are related by the following equation:

i = a*v*^b^

In this case, v represents the scan rate, and i is the peak current. The b value represents the slope in the log(*v*) vs log(i) plot. If the b value is 0.5, it suggests that ion diffusion is the dominant factor controlling the electrochemical behavior, whereas a b value of 1.0 indicates that surface capacitance plays the key role in governing the behavior. The calculated b values for peak 1 and peak 2 are 0.66 and 0.89, respectively (Figure S9), showing that the electrochemical kinetics of FeTiO_3_ is mainly governed by capacitive behavior.

**Figure 3:**
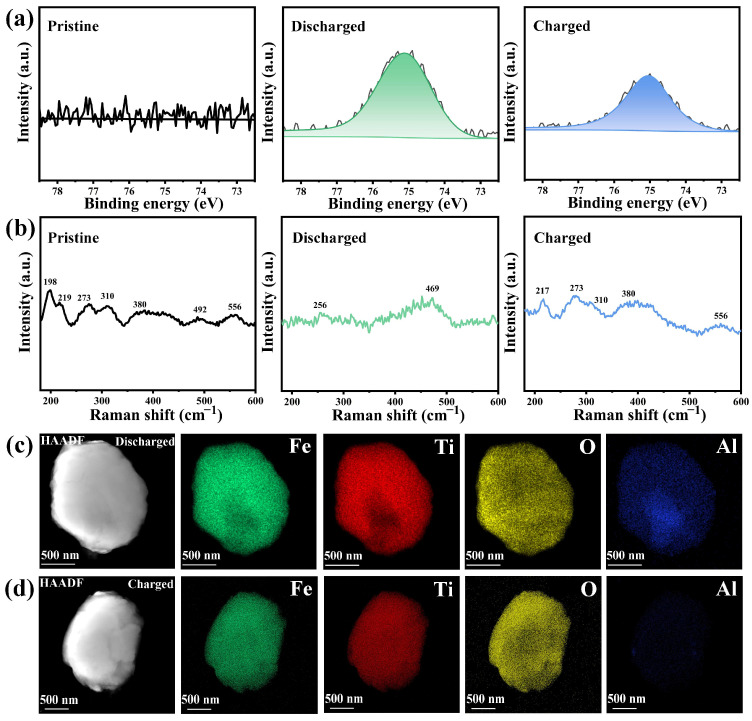
Electrochemical mechanism exploration towards lunar soil simulants-based batteries with FeTiO_3_ cathode via ex situ characterizations of (**a**) Ex situ XPS results for Al element in FeTiO_3_ at pristine, discharged, and charged states. (**b**) Ex situ Raman spectra of FeTiO_3_. (**c**,**d**) Ex situ TEM images of FeTiO_3_.

In the design of an in situ fabricated battery system based on lunar soil resources, FeTiO_3_ has demonstrated potential as a cathode material for AAIBs. To further explore the electrochemical behavior and structural changes of FeTiO_3_, we conducted ex situ XPS and ex situ Raman spectroscopy to reveal the chemical environment and crystal structure evolution under different charge and discharge states. Figure 3a displays the Al 2p XPS spectra of FeTiO_3_ at various charge–discharge states. The Al signal is stronger in the discharged state than in the charged state, indicating that Al^3+^ can reversibly intercalate into the FeTiO_3_ lattice. During the charging process, some aluminum ions deintercalate from the FeTiO_3_ structure, but some remain in the material. These residual aluminum ions may have strong interactions with active sites in the FeTiO_3_ lattice, preventing their complete deintercalation. This phenomenon may contribute to the capacity fade observed during cycling.

Comparing with the Raman spectra (Figure 3b), we can discern the structural changes in FeTiO_3_ during the aluminum-ion intercalation and deintercalation processes. In the pristine state, the Raman spectrum of FeTiO_3_ exhibits several characteristic peaks, with peaks at 198 and 219 cm^−1^ associated with the low-frequency vibrational modes of Fe-O-Ti bonds, reflecting the local bending vibrations of Fe and Ti atoms. The peaks at 273 and 310 cm^−1^ correspond to the bending vibrations of Fe-O bonds, particularly the internal bending vibrations of the oxygen octahedra surrounding Fe atoms, reflecting the symmetry and local environment of FeO_6_ octahedra in the FeTiO_3_ structure. The peak at 380 cm^−1^ is typically associated with the internal bending vibrational modes of TiO_6_ octahedra. The peaks at 492 and 556 cm^−1^ correspond to the symmetric and antisymmetric stretching vibrations of Ti-O bonds, reflecting the stable structure of TiO_6_ octahedra. In the discharged state, the intensity of most characteristic peaks in the pristine state significantly decreases, particularly the peaks at 273 cm^−1^, 310 cm^−1^, 380 cm^−1^, and 492 cm^−1^, which almost disappear. This suggests that during discharge, aluminum ions successfully intercalate into the FeTiO_3_ lattice, causing significant changes in the lattice structure, likely due to the rearrangement or disruption of Fe-O or Ti-O bonds following aluminum-ion intercalation. Additionally, a new peak appears at 469 cm^−1^, which may correspond to a new phase or compound, such as the formation of aluminum–oxygen bonds formed during the discharge process. These changes indicate that the FeTiO_3_ lattice undergoes rearrangement or distortion during discharge. In the charged state, some of the characteristic peaks from the pristine state reappear after charging, indicating partial recovery of the lattice structure as some aluminum ions deintercalate from the FeTiO_3_ lattice. However, the lattice structure does not fully return to its initial state, possibly due to incomplete deintercalation of some aluminum ions or irreversible structural changes.

Additionally, the ex situ high-angle annular dark-field (HAADF) images of FeTiO_3_ and the corresponding EDS mapping results show that, except for Al, all other elements are uniformly distributed during the charge and discharge states (Figure 3c,d). Compared to the discharged state, the density of the Al signal is lower after the battery is fully charged. This is consistent with the XPS and Raman results, further supporting our previous conclusions.

## 4. Conclusions

Overall, this work proposes for the first time the in situ fabrication of lunar-based batteries based on lunar soil simulants, and innovatively applies the novel FeTiO_3_ cathode material in AAIBs. Through facile treatment, we closely replicated the natural characteristics of ilmenite found in lunar soil. The newly fabricated lunar soil-based batteries demonstrated a capacity of 68.1 mAh g^−1^ at a high current density of 1.0 A g^−1^, with a capacity retention rate of 89.6% after 100 cycles. As research on lunar resources deepens, more sustainable, flexible, and scalable energy storage solutions are expected to emerge, capable of meeting the long-term energy needs of lunar bases while also being applicable to exploration missions on Mars, other planets, and asteroids. By adapting to the geological and resource characteristics of different celestial bodies, these technologies hold promise as a critical support for deep space exploration. Notably, the discovery of natural few-layer graphene in lunar soil unveils new potential for developing advanced battery materials and composite functional materials. Our work provides essential experimental support for the development of efficient energy storage materials based on lunar resources, and offers new directions for future lunar resource utilization and deep space exploration.

## Data Availability

Data are contained within the article and Supplementary Materials.

## Supplementary Materials

The following supporting information can be downloaded at: https://www.mdpi.com/article/10.3390/ma18030471/s1, Figure S1: SEM image of FeTiO_3_ before ball milling; Figure S2: XRD pattern of as-prepared FeTiO_3_ before ball milling; Figure S3: SEM and EDS elemental mappings of as–prepared FeTiO_3_; Figure S4: TEM image of as–prepared FeTiO_3_; Figure S5: Galvanostatic charge and discharge curve of as–prepared sample at a current density of 5.0 A g^−1^; Figure S6: SEM image of the as–prepared FeTiO_3_ electrode after cycling.; Figure S7: Long–term cycling performance of lunar soil simulants–based cathode at a current density of 5.0 A g^−1^; Figure S8: Nyquist plots of FeTiO_3_ electrode before and after cycling; Figure S9: (a) CV of FeTiO_3_ with different rates in the region of 0.30–1.70 V. (b) the matching plots of slope b of the redox peaks and log(peak current, i) vs. log(scan rate, v); Table S1. List of the abbreviations and their full Terms.
