# Supplementary material for: Construction of Lunar Soil Simulants-Based Aluminum-Ion Battery Systems
Source: Materials (Basel). 2025 Jan 21;18(3):471. doi: 10.3390/ma18030471 (PMC11818722; doi:10.3390/ma18030471)
Supplement: Supplementary file 1 [file materials-18-00471-s001.zip › materials-3348429-supplementary.pdf]

Supporting Information

Construction of Lunar Soil Simulants-Based Aluminum-Ion Battery Systems

*Shaokang Su, Jingzhen Li, Chunhao Sun, Kai Du, Chengjie Wang, Mingshan Han, Jing Geng, Yongde Long and Yuxiang Hu**

Key Laboratory of Advanced Functional Materials of Education Ministry of China, College of Materials Science and Engineering, Beijing University of Technology, Beijing, 100124, China.

ssk610050375@163.com (S.S.); jzli11@bjut.edu.cn (J.L.);
chunhaosun9808@163.com (C.S.); dukai@emails.bjut.edu.cn (K.D.);
wangchengjie@emails.bjut.edu.cn (C.W.); mshan@emails.bjut.edu.cn (M.H.);
b202377028@emails.bjut.edu.cn (J.G.); yongde_long@bjut.edu.cn (Y.L.)

Correspondence: y.hu@bjut.edu.cn

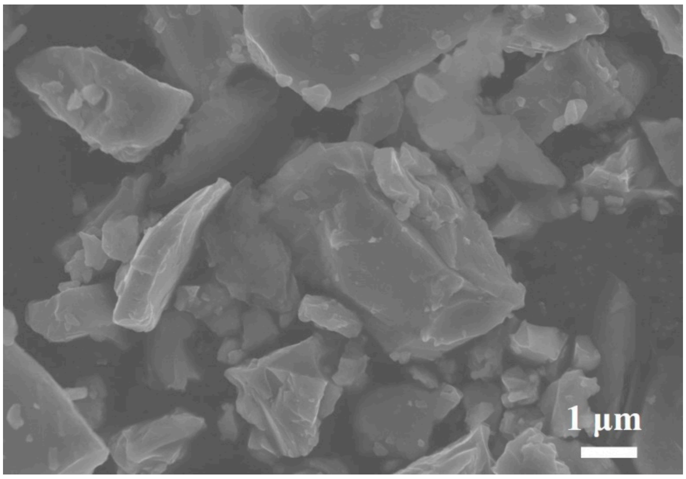

Figure S1. SEM image of $FeTiO_3$ before ball milling.

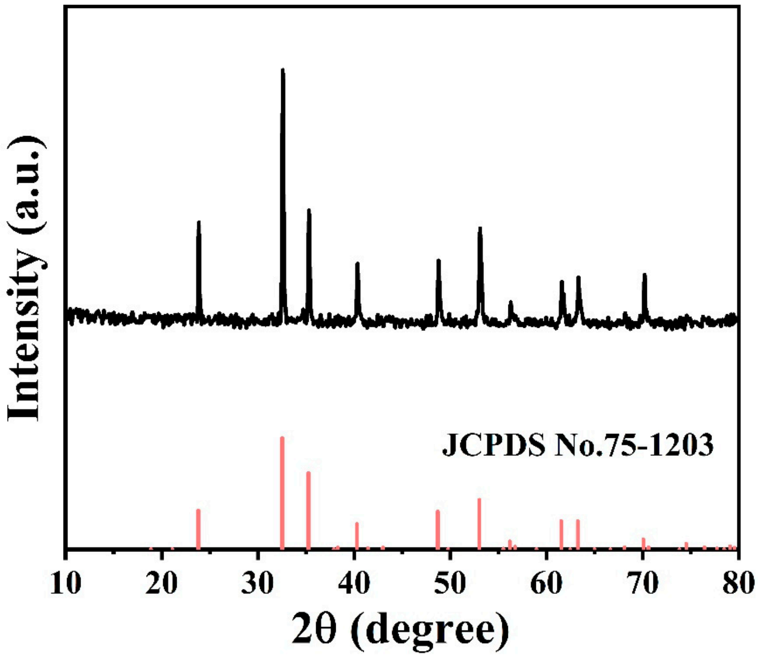

Figure S2. XRD pattern of as-prepared $FeTiO_3$ before ball milling.

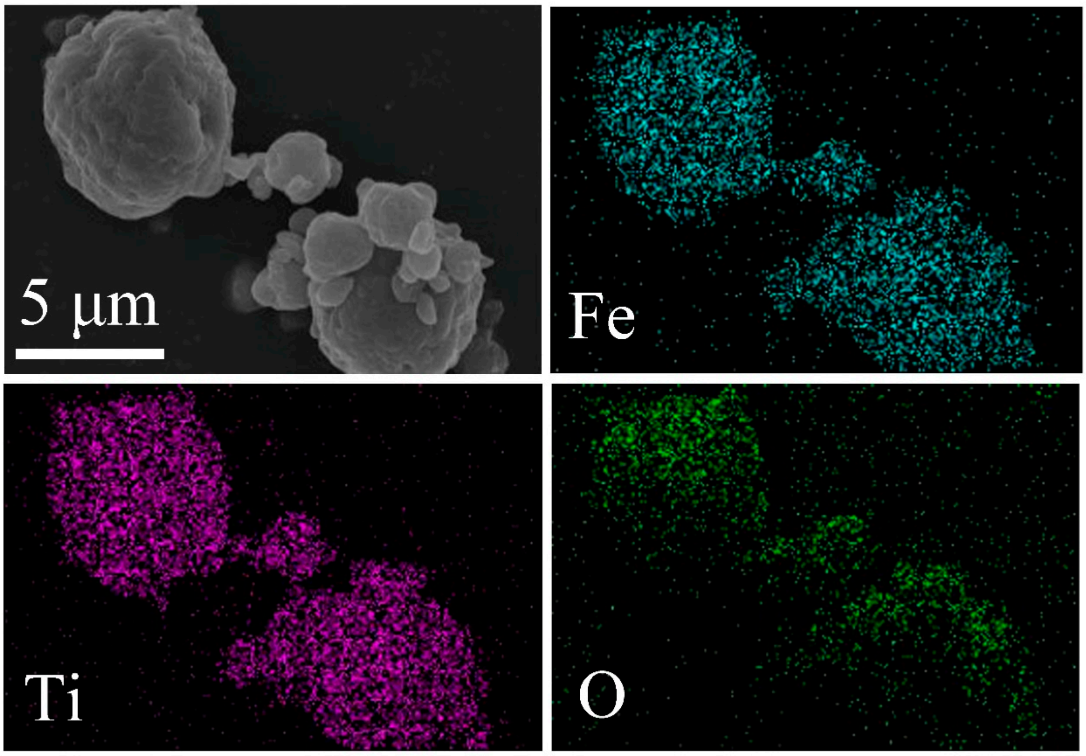

Figure S3. SEM and EDS elemental mappings of as-prepared $FeTiO_3$.

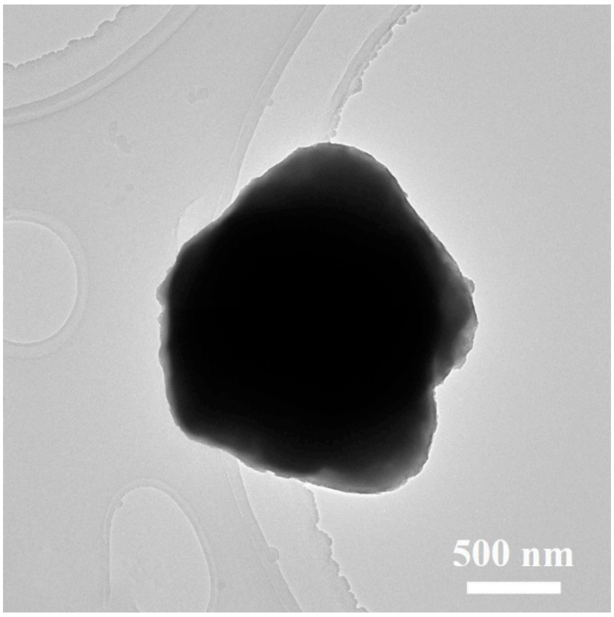

Figure S4. TEM image of as-prepared $FeTiO_3$.

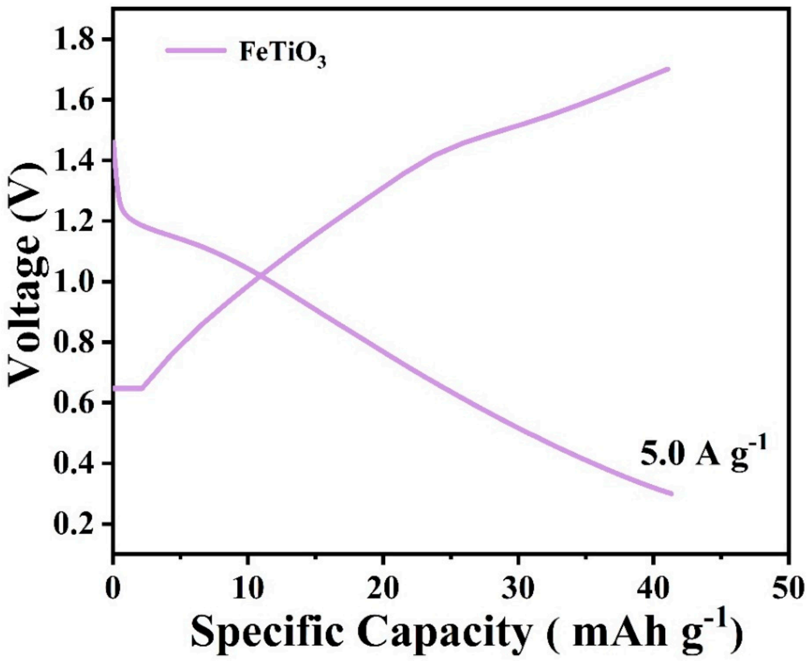

Figure S5. Galvanostatic charge and discharge curve of as-prepared sample at a current density of 5.0 A g^{-1}.

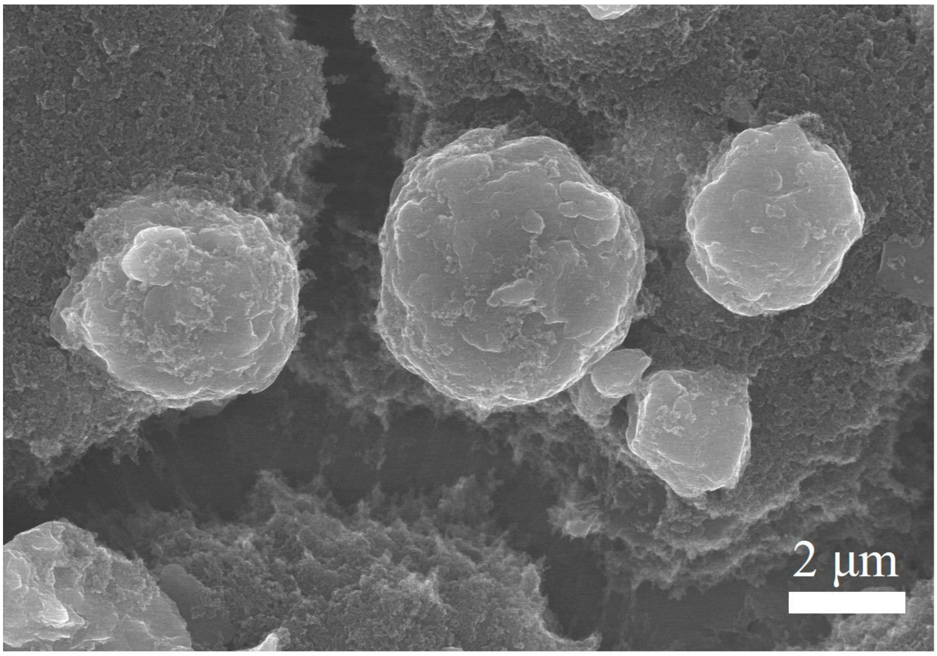

Figure S6. SEM image of the as-prepared $FeTiO_3$ electrode after cycling.

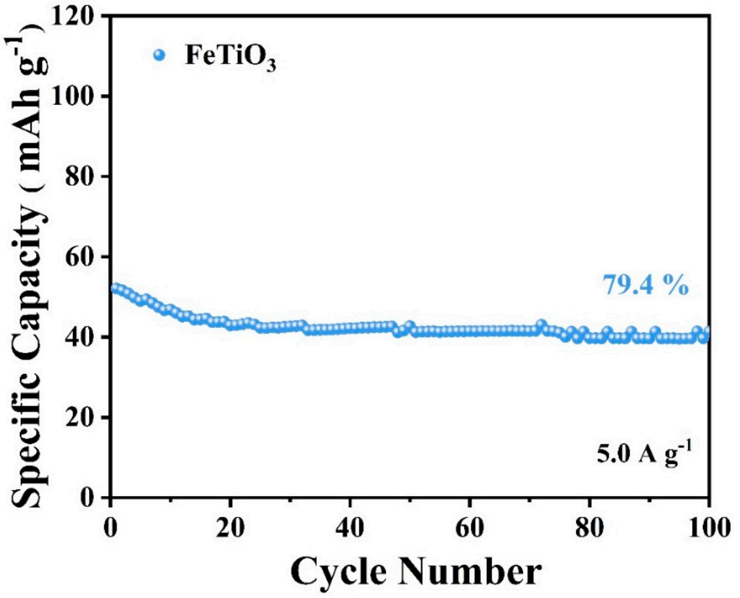

Figure S7. Long-term cycling performance of lunar soil simulants–based cathode at a current density of 5.0 A g^{-1}.

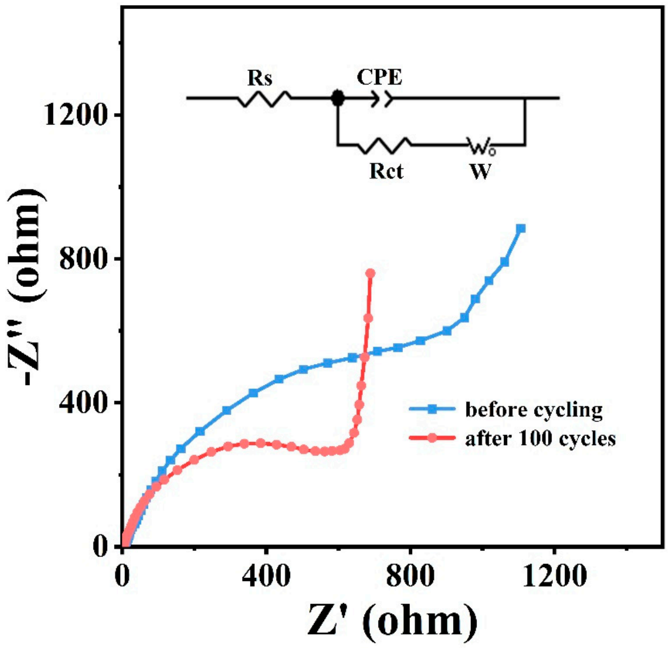

Figure S8. Nyquist plots of $FeTiO_3$ electrode before and after cycling.

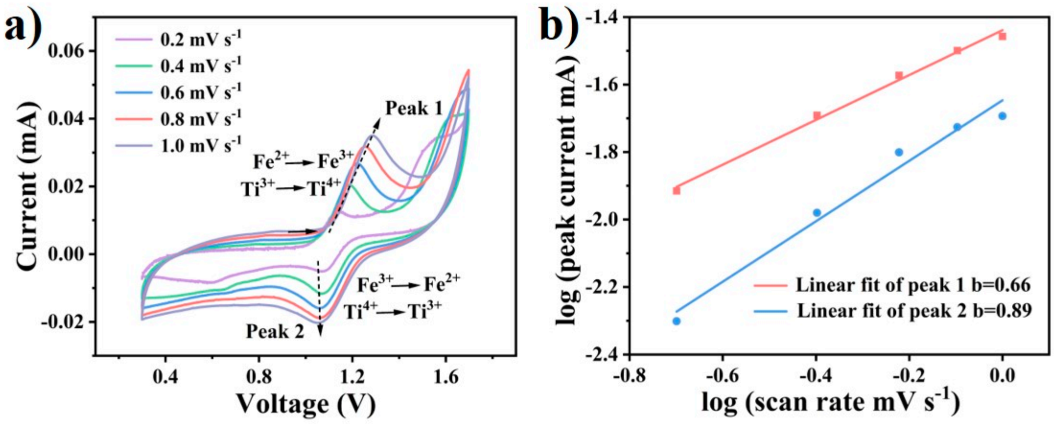

Figure S9. (a) CV of $FeTiO_3$ with different scan rates in the region of 0.30–1.70 V. (b) the matching plots of slope b of the redox peaks and log(peak current, i) vs. log(scan rate, *v*).

Table S1. List of the abbreviations and their full terms

Abbreviations	Full Terms
ISRU	In Situ Resource Utilization
AAIBs	Aqueous Aluminum-Ion Batteries
XRD	X-Ray Diffraction
XPS	X-Ray Photoelectron Spectroscopy
SEM	Scanning Electron Microscopy
TEM	Transmission Electron Microscopy
SAED	Selected Area Electron Diffraction
CV	Cyclic Voltammetry
GCD	Galvanostatic Charge-Discharge
EIS	Electrochemical Impedance Spectroscopy
EDS	Energy Dispersive X-Ray Spectroscopy
HAADF	High-Angle Annular Dark-Field
